# Most HIV DNA in PBMC is present in non-gut homing, resting memory CD4+ T cells with a ß7-CD38-CD127 high phenotype

**DOI:** 10.1186/1758-2652-13-S3-O2

**Published:** 2010-11-04

**Authors:** KK Koelsch, Y Xu, M Bailey, K McBride, N Seddiki, K Suzuki, J Murray, DA Cooper, AD Kelleher, J Zaunders

**Affiliations:** 1 University of New South Wales / National Centre in HIV Epidemiology and Clinical Research (NCHECR), Darlinghurst, Australia; 2 St Vincent’s Centre for Applied Medical Research, Darlinghurst, Australia; 3 University of New South Wales, NCHECR / Department of Mathematics, Kensington, Australia

## Background

Recent studies report that most CD4+ T cell depletion occurs in gut-associated lymphoid tissue (GALT), inferring that most viral replication occurs in these tissues. Memory CD4 T lymphocytes in peripheral blood comprise two main subsets: those with integrins α4ß7 that recirculate through GALT; and those with α4ß1 that do not access GALT. We tested the hypothesis that α4ß7+ CD4 T cells are preferentially infected with HIV DNA.

## Methods

Peripheral blood or leukopheresis packs were collected from a total of 11 patients: seven with untreated chronic HIV infection (CHI); two with primary HIV infection (PHI); and two with long-term fully suppressed CHI. CD4 T cells were first isolated by negative selection. Then further FACS sorted into highly purified subsets of CD3+CD4+CD45RO+ cells: ß7+ vs. ß7-; CD25+CD127dim Treg vs CD127high; CD27+ vs. CD27-; and CD38+ vs. CD38- subsets. DNA was extracted and total HIV DNA copies quantified by real-time Polymerase Chain Reaction.

## Results

Approximately 90% of HIV DNA copies in PBMC from the three groups were in CD3+CD4+CD45RO+ memory cells. Further subdivision of these memory CD4 T cells in early and/or untreated CHI found that a median 80% of this HIV DNA was found in ß7- non-gut homing cells. Similar results were obtained in PHI and in fully suppressed CHI. A median 8% of HIV DNA in early untreated CHI was found in highly purified Tregs, with the majority in CD127high memory cells. Only 9% of HIV DNA was found in CD38+ activated memory, while 32% was found in effector memory CD27- cells.

## Conclusions

Our results demonstrate that the majority of the HIV reservoir in PBMC is present in non-gut homing memory CD4 T cells with a resting CD127highCD38-CD27+ phenotype. These cells recirculate preferentially through secondary lymphoid tissue, but not GALT. These results are important for the design of therapy regimens targeting the HIV reservoir.

